# The Physicochemical Principles Governing Nicotine Delivery from Oral Nicotine Pouches

**DOI:** 10.3390/ma19204410

**Published:** 2026-10-20

**Authors:** Håkan Engqvist

**Affiliations:** Department of Materials Science and Engineering, Division of Applied Materials Science, Ångström Laboratory, Uppsala University, Box 524, 75120 Uppsala, Sweden; hakan.engqvist@angstrom.uu.se; Tel.: +46-702569500

**Keywords:** nicotine pouch, oral drug delivery, buccal absorption, nicotine release, water activity, moisture content, nicotine speciation, diffusion, matrix design, calcium sulfate

## Abstract

**Highlights:**

- Nicotine pouches are becoming a major and rapidly growing nicotine category worldwide.
- Nicotine content alone is insufficient to predict onset, peak exposure or total systemic uptake.
- Release and uptake reflect coupled hydration, pH evolution, diffusion and mucosal permeation.
- Dry and moist pouches differ in hydration, transport behaviour and release kinetics.
- Formulation design can tailor the onset, intensity and duration of nicotine delivery.

**Abstract:**

Nicotine pouches represent a rapidly expanding category of tobacco-free oral nicotine products. While these products are often compared primarily on the basis of nicotine content, evidence from in vitro dissolution/permeation studies and controlled clinical pharmacokinetic studies shows that nicotine delivery is governed by a complex interplay between physicochemical and formulation-related parameters. Moisture content, water activity, pH, nicotine speciation, matrix composition, particle morphology, and pouch architecture collectively influence nicotine dissolution, transport, and absorption through the oral mucosa. This review examines nicotine pouches from a pharmaceutical and drug-delivery perspective, treating them as engineered oral delivery systems rather than conventional consumer products. The mechanisms governing nicotine release are discussed in relation to acid–base chemistry, diffusion phenomena, hydration dynamics, and matrix design. Emphasis is also placed on the distinction between moist and dry pouch formulations and how these product families differ in hydration state, transport behavior, sensory properties, and release kinetics. The review further evaluates matrices, humectants, pH modifiers, nicotine sources, and emerging alternative carrier materials. Together, in vitro characterization and clinical PK data indicate that nicotine delivery depends on the coupled effects of water availability, matrix structure, nicotine release and local pH conditions. Understanding nicotine pouches as controlled oral delivery systems provides a framework for improved product characterization, more meaningful performance comparisons, and development of technologies with tailored pharmacokinetic profiles.

## 1. Introduction

Nicotine pouches have emerged as one of the fastest-growing categories of tobacco-free nicotine products worldwide [1]. Their rapid adoption has been accompanied by a growing body of analytical, in vitro dissolution/permeation and clinical pharmacokinetic research. Nevertheless, important mechanistic relationships between formulation properties, matrix structure, dynamic oral conditions and systemic nicotine exposure remain incompletely resolved, particularly when products with different carrier systems and hydration states are compared.

At first glance, nicotine pouches may appear deceptively simple. A small white pouch containing nicotine is typically placed between the upper lip and gingiva, where nicotine is released into saliva and absorbed across adjacent oral mucosa. This placement primarily exposes labial and gingival mucosa rather than the cheek (buccal) mucosa. These oral sites differ in epithelial thickness and degree of keratinisation; accordingly, “oral mucosal absorption” is used here as the broader term, while “buccal” is retained where used in the cited literature. Products are often compared on the basis of declared nicotine content, but dose alone does not capture these anatomical and formulation-dependent determinants of exposure.

Nicotine delivery from a pouch is governed by coupled physicochemical processes that begin immediately after placement in the oral cavity [2,3,4,5,6]. Saliva penetrates the pouch matrix, initiating hydration of the carrier and dissolution of soluble components. Nicotine then diffuses through the hydrated structure, partitions into the saliva phase and permeates the oral mucosa before entering systemic circulation. Pouch-specific characterization studies show substantial differences among commercial products in dissolution, particle size, density, water activity, pH and extract viscosity [7,8]. The matrix should therefore be considered part of the delivery system rather than simply a container: its composition and architecture influence fluid transport, dissolution and diffusion together with moisture, pH modifiers and other excipients.

The distinction between so-called *dry* and *moist* nicotine pouches is important. While these categories are frequently used in commercial marketing and consumer discussions, they are often poorly defined scientifically. Moisture content is only one component of a broader formulation landscape that includes hydration state, water mobility, matrix architecture, viscosity, and transport properties. Understanding how these factors interact is essential for explaining why products with similar nicotine contents may exhibit markedly different release profiles, sensory characteristics, and pharmacokinetic behavior [9]. The rapid development of nicotine pouch technology has also introduced new carrier systems and formulation approaches that extend beyond traditional cellulose-based matrices. Emerging materials, including engineered porous structures and inorganic delivery platforms, may provide opportunities for more precise control of nicotine release and absorption in future products. These developments further reinforce the need to view nicotine pouches through the lens of formulation science and controlled drug delivery.

**Figure 1:**
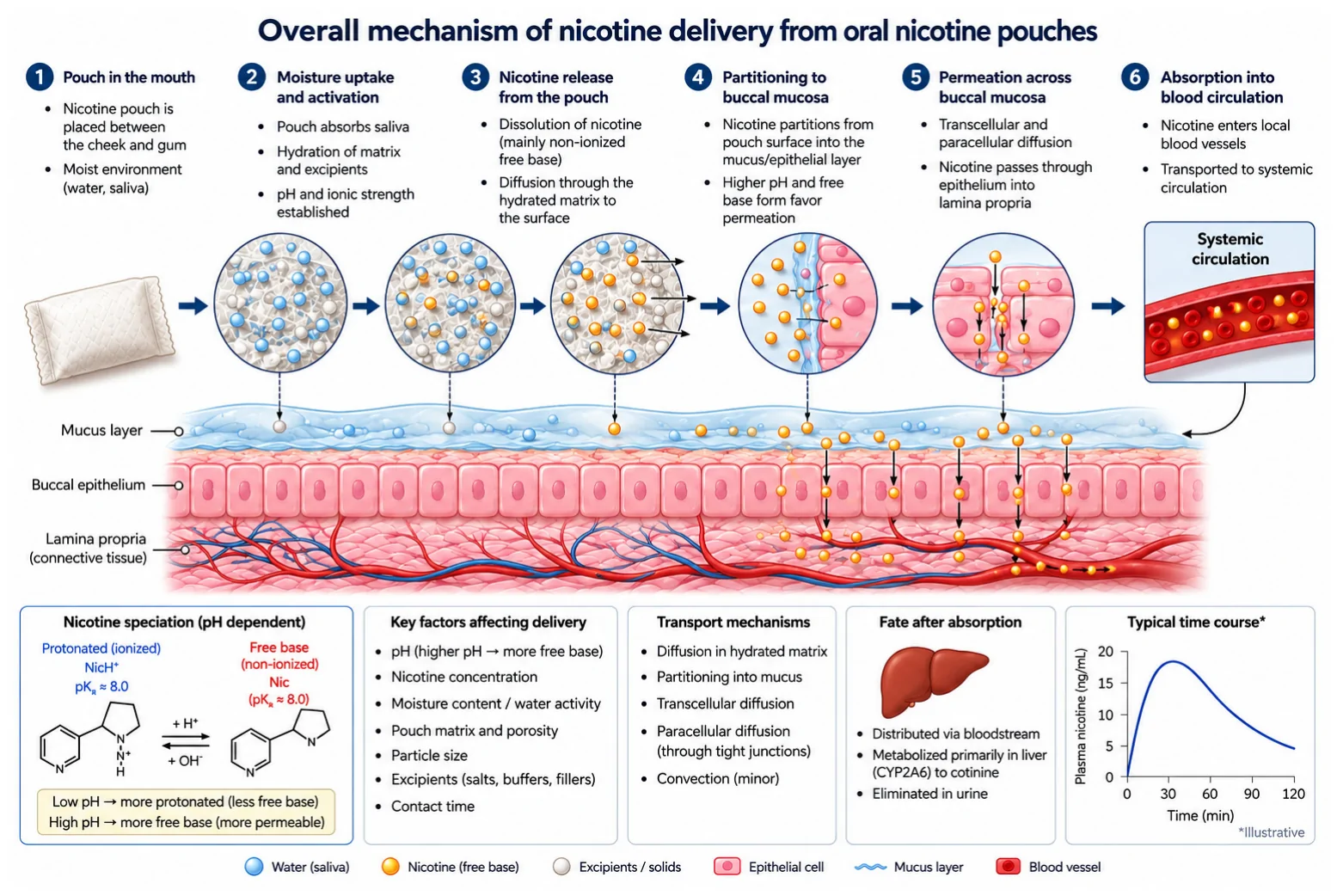
Mechanism of nicotine delivery from nicotine pouches. Schematic figure prepared by the author for this review using ChatGTP-6 from Open AI.

This review examines nicotine pouches as engineered oral nicotine delivery systems and provides a comprehensive overview of the physicochemical mechanisms that govern their performance. Emphasis is placed on nicotine chemistry, moisture and hydration dynamics, matrix design, diffusion-controlled release, and buccal absorption; see Figure 1. By focusing on the underlying scientific principles rather than nicotine content alone, the review aims to provide a framework for understanding current products and guiding the development of next-generation nicotine delivery technologies. Unlike reviews organized primarily around product categories or exposure outcomes, this review explicitly separates matrix release, transport through saliva, mucosal permeation and systemic pharmacokinetics, and identifies the measurements needed to connect these stages. This integrated materials-to-exposure framework is the specific contribution of the present review.

## 2. Physicochemical Properties of Nicotine

Nicotine is chiral. Tobacco-derived nicotine is predominantly the (S)-enantiomer, whereas synthetic nicotine can be racemic or enriched in (S)-nicotine. Stereoisomer composition and manufacturing route may affect impurity profiles and pharmacological response [10,11].

### 2.1. Nicotine Chemistry

Nicotine is commonly described as a weak dibasic alkaloid with pKa values of approximately 3.1 and 8.0 at room temperature; the lower value is associated with the pyridine nitrogen and the higher value with the pyrrolidine nitrogen [4,5]. Under pH conditions relevant to nicotine pouch use, the pyrrolidine pKa is the dominant equilibrium governing the proportion of monoprotonated and unprotonated nicotine.

The pharmacokinetic performance of nicotine pouches is fundamentally governed by the acid–base chemistry of nicotine. Unlike many active pharmaceutical ingredients that exist predominantly in a single ionic state under physiological conditions, nicotine can exist in multiple protonation states depending on the surrounding pH environment. This characteristic makes nicotine delivery highly sensitive to formulation variables such as pH modifiers, buffer capacity, moisture content, and saliva composition.

Nicotine is commonly described as a weak dibasic alkaloid with two pKa values of approximately 3.1 and 8.0. The lower pKa is associated with protonation of the pyridine nitrogen, whereas the higher pKa corresponds to the pyrrolidine nitrogen. Under the pH conditions relevant to nicotine pouch use, typically between pH 6 and pH 10, the pyrrolidine nitrogen largely determines the protonation state and transport properties of the molecule.

- Fully protonated nicotine (NicH_2_^2+^).
- At pH values encountered in nicotine pouches, the dominant equilibrium is between monoprotonated nicotine and unprotonated (free-base) nicotine. The neutral form is more lipophilic and generally crosses epithelial cell membranes more readily. Ionised nicotine should not, however, be considered completely non-permeating: hydrophilic species can contribute to transport through aqueous and paracellular pathways, although transcellular diffusion of uncharged nicotine is generally favored [12,13]. Thus, free-base fraction is an important determinant of absorption rate but not the sole determinant of total oral uptake.

At the pH values encountered in most nicotine pouches, the equilibrium is primarily between monoprotonated nicotine and free-base nicotine. The distinction between these forms is critical because they possess markedly different transport characteristics. The protonated form is highly water soluble and readily dissolves into saliva, but its positive charge limits passive diffusion across the lipid-rich cell membranes of the oral mucosa. In contrast, free-base nicotine is electrically neutral and significantly more lipophilic, enabling it to partition into biological membranes and diffuse across epithelial tissues much more efficiently. Consequently, the concentration of free-base nicotine rather than the total nicotine concentration is often the most important determinant of nicotine absorption rate. Two products containing identical amounts of nicotine may therefore produce substantially different pharmacokinetic profiles if their pH values differ [4,5].

The relative proportions of protonated and unprotonated nicotine are governed by the local pH environment. As the pH increases above the pKa of nicotine, the equilibrium progressively shifts toward the uncharged free-base form. Because free-base nicotine exhibits substantially greater membrane permeability than protonated nicotine, even modest increases in pH can significantly enhance nicotine absorption across the oral mucosa. This relationship can be quantitatively described by the Henderson–Hasselbalch equation [6], which relates the ratio of free-base to protonated nicotine to the pH of the surrounding environment:

$$\frac{\left[Nic\right]}{\left[NicH^{+}\right]}=10^{\left(pH-pK_{a}\right)}$$

where Nic represents unprotonated (free-base) nicotine and NicH^+^ represents protonated nicotine.

The fraction of nicotine present in the free-base form can therefore be expressed as:

$$f_{free}=\frac{\left[Nic\right]}{\left[Nic]+[NicH^{+}\right]}$$

which, after substitution, yields:

$$f_{free}=\frac{1}{1+10^{\left(pK_{a}-pH\right)}}$$

An approximate free-base nicotine fraction is calculated using the Henderson–Hasselbalch relationship with a pyrrolidine pKa of 8.0 at approximately 25 °C; see Table 1 [4,5,6].

This relationship is highly non-linear. Around the pKa of nicotine, relatively small changes in pH can produce dramatic changes in the proportion of membrane-permeable nicotine; see Table 1.

This table illustrates why pH has become one of the most important formulation parameters in modern nicotine pouches. Increasing pH from 7.0 to 9.0 increases the free-base fraction by approximately an order of magnitude. To achieve this effect, manufacturers commonly incorporate alkaline additives such as sodium carbonate, sodium bicarbonate, calcium carbonate, magnesium hydroxide, or other buffering agents. These ingredients raise the local pH within the pouch and saliva interface, thereby shifting the equilibrium toward the free-base form and enhancing buccal absorption.

In addition to pH, the chemical form and source of nicotine can influence handling, stability and dissolution. Nicotine may be incorporated as free base or as salts such as bitartrate, depending on product formulation. Nicotine polacrilex is not a nicotine salt; it is an ion-exchange resin complex used extensively in nicotine replacement products [14]. Product-specific identification of the exact nicotine salt is not consistently available in the public literature, so individual salts should not be assumed to be common in pouches without analytical or manufacturer-specific evidence.

The chemistry becomes even more complex when considering that the microenvironment surrounding the pouch may differ substantially from bulk saliva. As nicotine is released, local pH gradients can develop within the pouch matrix and at the pouch-mucosa interface. Furthermore, saliva production, salivary buffering capacity, oral fluid volume, and individual physiological differences can all influence nicotine speciation during product use [8].

In addition to pH, the chemical form of nicotine used in the formulation can influence release behavior. Commercial nicotine pouches commonly utilize nicotine salts such as nicotine bitartrate, nicotine lactate, nicotine citrate, nicotine malate, or nicotine polacrilex. These salts differ in their solubility, dissolution behavior, hygroscopicity, and interaction with matrix materials. Following dissolution, however, the absorption process remains governed primarily by the equilibrium between protonated and free-base nicotine [4,5].

From a drug delivery perspective, nicotine chemistry provides the mechanistic link between formulation design and clinical performance. Nicotine content determines the theoretical dose available within the pouch, but pH and nicotine speciation largely determine how much of that nicotine becomes membrane-permeable and available for rapid absorption. Understanding this relationship is therefore essential for interpreting differences between products and for designing next-generation nicotine delivery systems with predictable pharmacokinetic profiles.

In many respects, modern nicotine pouches can be viewed as miniature chemical reactors in which pH adjustment, hydration, dissolution, and nicotine speciation occur simultaneously during use. The resulting balance between protonated and free-base nicotine ultimately dictates the efficiency of buccal delivery and represents one of the most important scientific foundations of nicotine pouch technology.

### 2.2. Buccal Absorption

Following release from the pouch matrix, nicotine partitions into the saliva phase and is subsequently absorbed across the oral mucosa. However, buccal nicotine delivery is not governed by static physicochemical parameters. Instead, nicotine absorption is a dynamic process in which the local environment continuously evolves throughout pouch use [12,15].

Several interrelated variables contribute to this dynamic process, including:

- Local pH.
- Saliva production and saliva volume.
- Nicotine concentration in the saliva phase.
- Mucosal permeability.
- Pouch positioning and contact area.
- Duration of exposure.

Importantly, many of these variables are themselves time-dependent. The local pH typically does not immediately reach its equilibrium value when the pouch is placed in the oral cavity. Instead, alkaline buffering agents gradually dissolve, producing a progressive increase in pH and consequently an increasing fraction of free-base nicotine. Similarly, nicotine concentration within the saliva phase changes as dissolution, diffusion, absorption, and salivary clearance occur simultaneously.

The oral cavity therefore represents an evolving transport environment rather than a system at equilibrium. During the initial minutes of use, nicotine release may be primarily limited by hydration and dissolution processes. As hydration proceeds and pH rises, the fraction of membrane-permeable free-base nicotine may increase substantially, accelerating absorption. At later stages, depletion of nicotine from the pouch, dilution by saliva, and decreasing concentration gradients may become the dominant factors controlling nicotine flux.

Consequently, nicotine uptake from a pouch should be viewed as a coupled sequence of hydration, dissolution, pH evolution, diffusion, and mucosal transport processes occurring simultaneously over time. The resulting pharmacokinetic profile, including Cmax, Tmax, and overall nicotine exposure, reflects the integrated outcome of these dynamic phenomena rather than any single formulation parameter measured at equilibrium.

From a drug delivery perspective, nicotine pouches may therefore be regarded as a reaction-diffusion system in which both the driving force for absorption (free-base nicotine concentration) and the transport pathways continuously evolve during use [12,13].

## 3. Components of Nicotine Pouch Formulations

Free-base nicotine is used in some nicotine delivery systems, particularly transdermal formulations, but many oral nicotine-replacement products use nicotine polacrilex or nicotine salts rather than free base. Free-base nicotine is volatile and chemically reactive and can contribute to harsh sensory characteristics; its use therefore requires formulation strategies that control stability, handling and local exposure [4,5,14].

Nicotine salts may also be used in oral formulations. Protonation can reduce volatility and modify handling and dissolution behavior, but the exact nicotine form used in commercial pouches is not always publicly disclosed. For this reason, the discussion below distinguishes established oral nicotine forms from pouch-specific evidence.

Established oral nicotine forms include nicotine bitartrate and other organic-acid salts, while nicotine polacrilex is an ion-exchange resin complex rather than a salt [14]. Different counterions and carrier interactions can alter solubility, crystallinity, water sorption and dissolution; however, pouch-specific comparative data remain limited and generalizations about the relative prevalence of individual salts should be made cautiously.

After dissolution, nicotine absorption is governed by the evolving balance between protonated and unprotonated nicotine, local pH, saliva transport and the mucosal barrier. Thus, nicotine source is one component of the delivery system rather than an independent predictor of pharmacokinetics.

Each salt exhibits unique solubility, hygroscopicity, crystal structure, dissolution characteristics, and interactions with pouch excipients.

Among these, nicotine bitartrate is one of the most commonly used nicotine salts in commercial nicotine pouches. The salt exhibits high aqueous solubility, good stability, and relatively straightforward manufacturing characteristics. Upon hydration, nicotine bitartrate dissolves and releases protonated nicotine into the aqueous phase, after which local pH conditions determine the equilibrium between protonated and free-base nicotine. Nicotine citrate and nicotine lactate exhibit similar behavior but may differ in dissolution kinetics, buffering capacity, and sensory characteristics. Organic acid counterions may also contribute to taste perception and influence the local pH microenvironment during use [7,8].

An important distinction is that the nicotine salt itself does not determine nicotine absorption. Rather, the salt acts as a nicotine reservoir that dissolves upon hydration. Once dissolved, nicotine absorption remains governed by the equilibrium between protonated and free-base nicotine. Consequently, two products utilizing different nicotine salts may ultimately produce similar nicotine uptake if they generate comparable pH conditions and free-base nicotine fractions during use. The choice of nicotine source also affects product hydration dynamics. Nicotine salts vary substantially in hygroscopicity and water-binding capacity, which can influence pouch moisture content, water activity, storage stability, and release kinetics. These interactions become particularly important when comparing moist and dry pouch formulations, where the availability and mobility of water strongly influence dissolution and transport processes.

### 3.1. Carrier Systems for Nicotine Delivery

Most commercial nicotine pouches employ cellulose-based carrier systems, including microcrystalline cellulose, powdered cellulose and other plant-derived fibers [2,7]. Cellulose offers low cost, absorbency, processability and a long history of use as a pharmaceutical excipient. Importantly, cellulose matrices are not functionally identical or necessarily passive. MCC grade, particle size, packing density, porosity and fiber morphology can alter capillary wetting, water uptake, swelling and diffusion pathways. Commercial pouch characterization has demonstrated differences in particle-size distributions, bulk density, soluble fraction, viscosity and dissolution behavior among cellulose-containing products [7]. Other fillers and bulking agents reported in pouch formulations include gums, maltitol and inorganic salts; their functions depend on the specific product composition [2]. Calcium sulfate has been evaluated as an alternative inorganic carrier for nicotine pouches [16]. In the studied system, calcium sulfate hemihydrate was hydrated to calcium sulfate dihydrate, forming a porous mineral matrix in which nicotine was incorporated during setting. Because publicly available independent evidence of broad commercial use remains limited, calcium sulfate is treated here as an evaluated emerging carrier platform rather than an established market-wide alternative.

Mechanistically, a set calcium sulfate matrix provides a porous mineral scaffold and reservoir for incorporated nicotine. Interconnected porosity may facilitate hydration and capillary transport, but the observed PK difference cannot be attributed to matrix architecture alone because pH, nicotine form, particle structure and other formulation variables also differed between products [16,17,18].

Future work should therefore establish quantitative relationships between matrix architecture, pore structure, water transport, release and pharmacokinetics across both organic and inorganic carriers.

The successful implementation of calcium sulfate-based pouches challenges the long-standing assumption that cellulose fibers are the preferred carrier material for oral nicotine delivery. Instead, the choice of matrix material itself emerges as an important design variable alongside nicotine concentration and pH. Porous inorganic matrices may therefore offer new opportunities to tailor nicotine pharmacokinetics through rational materials engineering, including control over hydration dynamics, liquid transport, nicotine distribution, and release kinetics.

Future studies should seek to establish quantitative relationships between matrix architecture, pore structure, water transport and nicotine pharmacokinetics. Such work may ultimately enable the design of nicotine pouches with predictable and tunable delivery profiles based not only on formulation chemistry but also on engineered transport properties.

#### Future Carrier Systems

The next generation of nicotine pouch technologies may extend beyond cellulose and calcium sulfate. However, the evidence base differs substantially between candidate materials, and a distinction should be made between materials demonstrated in nicotine delivery and materials whose relevance is inferred from broader drug-delivery research. Calcium sulfate has been evaluated directly in nicotine pouches, whereas several other inorganic systems remain prospective for this specific application.

Mesoporous silicate materials provide one of the clearest examples of an inorganic system investigated directly for nicotine loading and release. Ijbara et al. loaded nicotine into MCM-41 mesoporous silicates with different pore sizes and reported nicotine loading of approximately 17% for one formulation, with near-complete release from selected materials within 8 h in phosphate buffer [19]. Other studies have demonstrated that nicotine adsorption to periodic mesoporous organosilicas depends on surface chemistry, hydrophobicity, and the physicochemical environment [20]. These findings establish that pore architecture and surface chemistry can influence nicotine–material interactions, although performance, safety, sensory properties, manufacturability, and release kinetics in oral nicotine pouches have not yet been established.

Zeolites provide another example of porous inorganic materials with experimentally demonstrated nicotine interactions. Adsorption studies using BEA, MFI, HEU, and USY zeolites have shown substantial nicotine uptake from aqueous solutions and a dependence of adsorption behavior on zeolite structure and surface properties [21,22,23]. These studies were primarily directed toward adsorption and environmental removal rather than oral delivery. They therefore support the physicochemical feasibility of nicotine binding to zeolitic materials, but not their suitability as nicotine pouch carriers.

Ion-exchange resins occupy a different position because they are already established in oral nicotine delivery. Nicotine polacrilex is a complex of nicotine with a weak carboxylic cation-exchange resin and is widely used in nicotine replacement products [14,24]. The underlying principle—reversible ionic association followed by release in an aqueous ionic environment—demonstrates that carrier-nicotine interactions can be deliberately used to modify nicotine availability. Application of such systems within modern pouch formulations is therefore plausible, but pouch-specific performance depends on formulation, resin loading, ionic conditions, pH, and product architecture.

Other materials, including calcium phosphates and biodegradable polymers such as PLGA, are well established as drug-delivery materials [25,26]. Their porosity, surface chemistry, degradation behavior, and capacity to incorporate active compounds make them conceptually relevant to future oral nicotine delivery. At present, however, evidence supporting their use specifically as nicotine pouch carrier matrices is limited. They should therefore be regarded as candidate materials rather than established nicotine-delivery platforms.

One further matrix-related parameter that warrants investigation is carrier bulk density. For a fixed pouch mass, carrier density influences pouch volume and geometry and therefore the contact area and nicotine loading per unit area presented to the oral mucosa. The relationship between these geometric parameters, nicotine release, and systemic uptake has not yet been adequately characterized.

### 3.2. Additives for Mouth Feel and Nicotine Delivery

#### 3.2.1. Humectants

Humectants represent a critical but often overlooked component of nicotine pouch formulations. Although frequently regarded simply as moisture-retaining additives, humectants play a multifaceted role in determining product stability, hydration dynamics, nicotine release kinetics, sensory perception, and overall product performance.

The most commonly used humectants in nicotine pouches are glycerol (glycerin) and propylene glycol, either individually or in combination [2]. Both compounds are highly hygroscopic and readily interact with water molecules through hydrogen bonding. As a result, they influence not only the total moisture content of the pouch but also the mobility and availability of water within the matrix.

From a formulation perspective, humectants serve several important functions. During manufacturing and storage, they help prevent excessive drying and maintain product softness. This contributes to pouch flexibility, handling properties, and consumer acceptance. In addition, humectants reduce moisture fluctuations during storage by buffering against changes in ambient humidity.

Glycerol is commonly reported as a humectant in nicotine pouch formulations, while propylene glycol is used in some oral and tobacco-related formulations; product-specific concentrations are not consistently disclosed in the peer-reviewed pouch literature [2,7]. Rather than assuming a universal concentration range, studies should report measured or declared humectant content when interpreting water activity and release.

An important distinction must therefore be made between moisture content and water activity. Two nicotine pouches may contain identical amounts of water yet exhibit significantly different release characteristics if their humectant compositions differ. Water strongly associated with glycerol or propylene glycol may be less available for dissolution and diffusion processes than free water present within the matrix.

Humectants also influence nicotine stability. Nicotine is susceptible to oxidation and degradation reactions that may occur during long-term storage. By regulating moisture distribution and microenvironmental conditions, humectants can contribute to improved shelf-life and product consistency. The concentration and type of humectant may further affect nicotine absorption indirectly through their influence on saliva interactions. More highly hydrated formulations generally require less saliva uptake before dissolution begins, whereas drier systems depend more heavily on saliva penetration and hydration kinetics [8].

From a sensory perspective, humectants contribute significantly to mouthfeel. Products containing higher levels of glycerol often exhibit a smoother, softer, and more lubricated sensation, whereas formulations with lower humectant content may feel drier and firmer during the initial stages of use.

Future formulation strategies may increasingly exploit humectants as active design tools rather than simple processing aids. By tailoring water activity, hydration kinetics, and matrix rheology, humectants may provide a powerful means of controlling nicotine release, mouthfeel and pharmacokinetic performance. Nicotine can undergo oxidation during storage, producing compounds including nicotine-N-oxide and other oxidation/degradation products; the rate depends on oxygen, light, temperature, pH and formulation environment [4,5]. Humectants may influence these reactions indirectly by changing water mobility and local microenvironment, but pouch-specific evidence remains limited.

#### 3.2.2. pH Modifiers

Among all formulation variables in nicotine pouches, pH modifiers arguably exert the greatest influence on nicotine absorption. While nicotine content determines the theoretical amount of nicotine available within a pouch, pH largely determines how much of that nicotine is present in the membrane-permeable free-base form capable of rapid buccal absorption. Consequently, pH modification represents one of the most important formulation strategies employed in modern nicotine pouch technology [6,8].

Humectants also influence texture and lubrication, but sensory effects depend on the full formulation and should not be attributed to glycerol or propylene glycol alone. Product-specific sensory studies are needed to link humectant concentration with perceived softness, dryness or mouthfeel.

The choice of alkaline compound can also influence release behavior. Highly soluble compounds such as sodium carbonate may generate rapid pH increases and accelerated nicotine uptake. Less soluble materials such as calcium carbonate may produce more gradual pH changes and potentially more sustained absorption profiles. In addition to influencing nicotine speciation, pH modifiers may affect several secondary formulation characteristics. Elevated pH can alter flavor perception, influence sweetness intensity, modify cooling sensations, and affect the stability of flavor compounds and nicotine itself.

Excessive alkalinity may also produce adverse sensory effects. Local irritation, burning sensations, mucosal discomfort, and tissue responses have all been associated with highly alkaline formulations. Consequently, pH optimization requires balancing nicotine delivery efficiency against product tolerability and user acceptance.

Future nicotine delivery systems may employ increasingly sophisticated buffering technologies capable of controlling not only the final pH but also the temporal evolution of pH during use. Such approaches could enable more precise control of nicotine release kinetics and pharmacokinetic profiles.

The effect of pH modifiers is dynamic rather than necessarily instantaneous. Saliva penetration, dissolution of alkaline components and salivary buffering occur concurrently, so the local pH at the pouch–saliva–mucosa interface may evolve during use. Direct time-resolved in-use pH data remain limited; therefore, differences between initial and later pH should be regarded as a mechanistic expectation that requires product-specific measurement rather than a universal feature of all pouches.

The choice of alkaline compound can influence buffering and dissolution behavior, but simple solubility arguments alone are insufficient to predict in-use nicotine delivery. Sodium carbonate is highly water soluble, whereas calcium carbonate is sparingly soluble; nevertheless, particle size, loading, matrix distribution, saliva volume and buffer capacity determine the actual pH-time profile. Comparative pouch studies are needed before assigning specific PK profiles to individual pH modifiers.

#### 3.2.3. Flavors

Modern nicotine pouches typically contain highly sophisticated flavor systems comprising dozens of individual compounds. These systems may include natural extracts, synthetic aroma compounds, cooling agents, sweeteners, acidity regulators, and flavor enhancers that collectively create the overall sensory experience. The most common flavor categories include mint, menthol, fruit, citrus, berry, coffee, spice, beverage-inspired profiles, and confectionery flavors. However, the sensory profile perceived by the user arises not from individual ingredients but from complex interactions among multiple compounds released over time [1].

Flavor release is itself a dynamic process governed by hydration and mass transport. As saliva penetrates the pouch matrix, flavor compounds dissolve and diffuse toward the pouch surface. Compounds with high water solubility are often released rapidly, whereas more hydrophobic components may exhibit slower and more sustained release profiles. Cooling agents deserve particular attention because they are widely used in modern nicotine pouches. Menthol, WS-3, WS-5, WS-23, and related compounds activate cold-sensitive TRPM8 receptors, creating a cooling sensation independent of actual temperature change. These cooling sensations may profoundly influence product perception. Cooling compounds can reduce perceived harshness, mask irritation associated with nicotine and alkaline additives and create the impression of smoother or more pleasant nicotine delivery. Sweeteners represent another important component of flavor systems. Common sweeteners include acesulfame potassium, sucralose, xylitol, sorbitol, and other polyols. Beyond sweetness, these ingredients may influence salivation, dissolution behavior, and mouthfeel. Organic acids such as citric acid, malic acid, tartaric acid, and lactic acid are often incorporated to modify flavor profiles and create desired sensory contrasts. However, these acids may simultaneously influence local pH conditions and interact with buffering systems used to enhance nicotine absorption.

Consequently, flavor systems should not be regarded as passive additives layered onto a nicotine formulation. Rather, they function as integrated components of the overall delivery system that influence hydration, salivation, pH development, sensory perception, user behavior, and potentially nicotine uptake itself. Future flavor systems may increasingly be engineered to provide temporal release profiles in which sweetness, cooling sensations, flavor intensity, and nicotine delivery evolve in a coordinated manner throughout pouch use. Such systems would further blur the distinction between flavor technology and drug delivery design.

Flavor systems influence sensory perception and product acceptance and may also modify salivation or local physicochemical conditions. Direct evidence that flavoring materially changes systemic nicotine uptake from pouches remains limited. Menthol and other compounds can affect epithelial transport in experimental systems, but such effects should be treated as formulation-specific hypotheses unless demonstrated in pouch dissolution-permeation or clinical PK studies [27].

### 3.3. Moisture as a Functional Design Parameter

Moisture represents one of the most important yet least understood formulation variables in nicotine pouch technology. Commercial products are often classified simply as either *dry* or *moist*, suggesting that water content is merely a descriptive product characteristic. From a pharmaceutical perspective, however, moisture should instead be regarded as a dynamic formulation parameter that fundamentally governs nicotine release, transport, and ultimately systemic nicotine exposure [7].

Unlike conventional oral dosage forms that disintegrate rapidly after administration, nicotine pouches remain structurally intact throughout use. Consequently, water serves not only as the dissolution medium for nicotine but also as the driving force for virtually every physicochemical process occurring within the pouch. Hydration determines the dissolution of nicotine salts, activation of buffering agents, swelling of the carrier matrix, diffusion of dissolved compounds, flavor release, and interactions with the oral mucosa. These processes occur simultaneously and continuously evolve during pouch use, making hydration one of the principal determinants of product performance.

Initial water content can affect hydration rate, nicotine dissolution, activation of pH modifiers, matrix swelling, diffusion, flavor release and mechanical softness. These variables are strongly interdependent, and pouch-specific characterization shows that moisture content should be interpreted together with water activity, density, soluble fraction and viscosity rather than as a stand-alone predictor [7].

For dry pouches, saliva penetration, matrix wetting/swelling, dissolution of nicotine and pH modifiers, pH evolution, diffusion and mucosal absorption begin on overlapping time scales rather than as a strictly sequential cascade. The relative rate of each process determines which step is rate-limiting at a given time. Moist products start with pre-distributed water and therefore require less external hydration before dissolution can proceed, but higher initial moisture does not necessarily guarantee faster systemic delivery because viscosity, water binding, pH and matrix transport also contribute [7,28].

### 3.4. Hydration Dynamics

Hydration begins immediately after placement of the pouch under the lip. Saliva penetrates the porous structure by capillary action, progressively wetting the carrier matrix and dissolving soluble formulation components.

Water activity (aw) describes the thermodynamic availability of water and complements gravimetric moisture content. In pouch systems, humectants, salts, matrix composition and pore structure can change water mobility even at similar total water contents. Pouch-specific studies have now reported water activity together with dissolution and other physical properties, supporting its use as a characterization parameter [7]. However, evidence that aw alone predicts clinical nicotine delivery is not yet sufficient; it should be interpreted as one variable within the coupled hydration and transport system.

Commercial nicotine products are generally described according to moisture content expressed as percentage water by weight. Although useful for manufacturing control, moisture content provides limited information regarding the physicochemical state of water within the formulation. Water activity (aw) is a thermodynamic parameter describing the availability of water for chemical and transport processes. It reflects how strongly water molecules interact with surrounding excipients rather than how much water is present. This distinction is particularly important for nicotine pouches [29,30,31].

Dry formulations depend more strongly on saliva uptake to establish a continuous aqueous phase, whereas moist formulations begin from a more hydrated state. This difference can influence early dissolution and sensory perception, but clinical delivery remains product-specific. Storage of high-moisture formulations may also require attention to nicotine redistribution, physical stability and microbiological control; these aspects are not adequately characterized in the current pouch literature and should be reported explicitly in future studies.

Hydration affects texture, softening, flavor release, cooling perception and the likelihood of saliva leakage. Dry products often begin firmer and hydrate during use, whereas moist products are softer at placement and may release soluble sensory components earlier. These are formulation tendencies rather than universal rules, and perceived “strength” should not be assumed to reflect systemic nicotine exposure.

Water activity may therefore represent a more meaningful predictor of nicotine delivery than moisture. Future studies should routinely report both parameters.

Nicotine release reflects coupled hydration, dissolution and diffusion. As hydration proceeds, liquid-filled transport pathways develop and concentration gradients evolve; later, depletion of releasable nicotine reduces the driving force. Pouch dissolution profiles are commonly compared empirically, and future work should test whether models such as Higuchi, Korsmeyer–Peppas or Weibull adequately describe specific pouch datasets rather than importing models from swelling HPMC tablets without validation. Matrix properties such as pore size, tortuosity, permeability, particle morphology and swelling behavior should be related directly to fitted release parameters.

Engineering a pouch for rapid onset, sustained delivery or a biphasic profile should therefore be presented as a design objective rather than an established consequence of any single material property. Demonstrating such control requires matched in vitro release/permeation data and clinical PK confirmation.

Hydration also strongly influences the sensory experience associated with nicotine pouch use. Dry formulations generally exhibit:

- Firmer texture.
- Slower softening.
- Delayed flavor release.
- Reduced drip.
- Greater dependence on individual saliva production

Moist formulations generally provide:

- Immediate softness.
- Rapid flavor release.
- Faster cooling sensations.
- Quicker nicotine perception.
- Increased risk of saliva leakage.

Importantly, user perception of “strength” is not necessarily correlated with nicotine dose. Rapid flavor release, cooling compounds, and immediate hydration may collectively create the impression of stronger nicotine delivery despite similar pharmacokinetic performance.

The overall release profile of a nicotine pouch emerges from the interaction between hydration, dissolution, diffusion, and absorption. Initially, nicotine release is often limited by saliva penetration and dissolution. As hydration progresses, diffusion through the liquid-filled pore network becomes increasingly important. At later stages, depletion of nicotine and decreasing concentration gradients gradually reduce release rates [32].

Consequently, nicotine release should be regarded as a hydration-controlled process rather than a simple diffusion phenomenon. Matrix properties including pore size, tortuosity, permeability, particle morphology, and swelling behavior all influence this process. Future formulations may deliberately manipulate hydration dynamics to achieve specific pharmacokinetic objectives. Rather than maximizing release rate, carrier systems could be engineered to provide rapid onset and sustained delivery. Hydration should therefore be regarded as the master variable governing nicotine pouch performance. By controlling how water enters, distributes within, and moves through the pouch, the formulation indirectly regulates virtually every subsequent process involved in nicotine delivery, including dissolution, pH evolution, free-base formation, diffusion, and buccal absorption.

## 4. Analytical Characterization of Nicotine Pouches

A fundamental challenge in nicotine pouch research is the lack of standardized analytical methods capable of fully characterizing product performance. While nicotine content is routinely reported by manufacturers and regulatory agencies, nicotine delivery is governed by a complex interplay between chemical, physical, and transport-related parameters that are substantially more difficult to quantify. As nicotine pouches increasingly resemble engineered oral delivery systems rather than simple consumer products, analytical characterization must extend beyond nicotine concentration alone. Comprehensive evaluation requires characterization of the active ingredient, carrier matrix, hydration behavior, pH evolution, transport phenomena, and pharmacokinetic performance.

### 4.1. Nicotine Quantification

Nicotine content is typically determined using high-performance liquid chromatography (HPLC), gas chromatography (GC), or liquid chromatography coupled to mass spectrometry (LC-MS). HPLC remains the most widely used technique due to its robustness, sensitivity, and suitability for routine quality control. Sample preparation generally involves extraction of nicotine from the pouch matrix followed by chromatographic separation and UV or mass spectrometric detection [33].

However, total nicotine content provides only limited information regarding product performance. Two products containing identical nicotine levels may exhibit substantially different release profiles and pharmacokinetic behavior. Consequently, future analytical approaches should increasingly focus on measuring nicotine availability and release kinetics rather than total nicotine content alone.

### 4.2. Determination of Nicotine Speciation

The ratio between protonated and free-base nicotine is one of the most important determinants of nicotine absorption. Nevertheless, direct quantification of nicotine speciation remains analytically challenging [33,34,35]. Current approaches typically estimate free-base nicotine fractions indirectly using pH measurements combined with Henderson–Hasselbalch calculations. While useful, such methods assume equilibrium conditions and may not accurately reflect the dynamic microenvironment present during pouch use. More advanced approaches include nuclear magnetic resonance (NMR) spectroscopy, headspace analysis, and chromatographic methods capable of distinguishing protonation states under controlled conditions. Future analytical development should focus on techniques capable of monitoring nicotine speciation in real time during hydration and use.

### 4.3. Moisture Content and Water Activity

Moisture content is routinely reported for nicotine pouches and is commonly determined using gravimetric drying methods, Karl Fischer titration, or thermogravimetric analysis (TGA). However, moisture content alone provides an incomplete description of water behavior within the formulation. Water activity measurements may provide significantly greater insight into hydration dynamics, nicotine mobility, flavor release, and product stability. Water activity reflects the thermodynamic availability of water rather than the total amount of water present and may therefore be more relevant to understanding release mechanisms. The simultaneous determination of moisture content and water activity is likely to become increasingly important for future product characterization [30].

### 4.4. pH and Buffering Capacity

Most published studies report pH values obtained after extraction of pouch contents into water. While such measurements provide useful comparative information, they may not accurately represent the transient pH conditions present during actual use. The pH environment experienced by nicotine continuously evolves as hydration proceeds and buffering agents dissolve. Consequently, equilibrium measurements may fail to capture the dynamic pH profile governing nicotine absorption. Future studies should therefore focus not only on equilibrium pH but also on pH evolution over time and formulation buffering capacity. Time-resolved pH measurements may prove particularly valuable when comparing moist and dry formulations or evaluating novel carrier systems [6].

### 4.5. Matrix Characterization

The carrier matrix represents the structural framework controlling hydration, transport, and release. Characterization of matrix architecture therefore plays a critical role in understanding product performance. Scanning electron microscopy (SEM) is particularly useful for visualizing fiber morphology, particle distribution, crystal structure, and pore architecture. For cellulose-based systems, SEM can reveal fiber orientation, packing density, and pore connectivity. For calcium sulfate-based systems, SEM enables visualization of crystal morphology and the interconnected pore network responsible for liquid transport [7].

Additional techniques include:

- Mercury intrusion porosimetry.
- Gas adsorption measurements (BET analysis).
- X-ray microtomography.

### 4.6. Dissolution and Release Testing

Physiologically relevant release testing remains a major analytical challenge. In addition to pouch-specific artificial-saliva and dissolution-permeation systems, established pharmaceutical tools can inform method development. Relevant approaches include the Ph. Eur. 2.9.25 chewing apparatus developed for medicated chewing gum, USP Apparatus 4 flow-through cells, and Franz diffusion cells for membrane permeation. None reproduces the pouch environment; method selection should therefore reflect the question being addressed, fluid volume, flow, mechanical compression, pH, membrane choice and sampling time [7,8].

Future platforms may integrate controlled artificial saliva flow, limited fluid volumes, mechanical contact and a mucosal or membrane compartment. Microfluidic oral models and real-time imaging could complement rather than replace established dissolution and diffusion methods.

- Microfluidic oral cavity models.
- Simulated mucosal interfaces.
- Real-time dissolution imaging.

Such systems may provide more meaningful predictions of nicotine release behavior and pharmacokinetic performance.

### 4.7. Toward Predictive Characterization

The future of nicotine pouch analytics lies in integrating chemical analysis, materials characterization, transport measurements, and pharmacokinetic modeling into unified predictive frameworks. Rather than treating nicotine content, pH, moisture content, and release behavior as isolated parameters, future approaches should seek to establish mechanistic relationships linking material properties to nicotine exposure. Such analytical frameworks may ultimately enable rational formulation design, reduce dependence on empirical testing, and accelerate the development of next-generation nicotine delivery systems.

## 5. Pharmacokinetics of Nicotine Pouches

The ultimate objective of any nicotine delivery system is to achieve controlled systemic exposure to nicotine. While the physicochemical properties of a pouch determine nicotine release and absorption mechanisms, the clinical performance of the product is ultimately reflected in its pharmacokinetic profile. Pharmacokinetic measurements therefore provide the critical link between formulation design and user exposure [36].

Unlike combustible cigarettes, where nicotine delivery occurs primarily through pulmonary absorption and reaches the systemic circulation within seconds, nicotine pouches rely on buccal absorption through the oral mucosa. The pharmacokinetic performance of nicotine pouches is commonly characterized using four principal parameters:

- Maximum plasma concentration (Cmax).
- Time to maximum concentration (Tmax).
- Area under the plasma concentration–time curve (AUC).

Tmax emerges from overlapping hydration, dissolution, pH evolution, matrix diffusion, saliva transport and mucosal permeation. These processes occur concurrently, and their relative kinetics differ among formulations. The published PK studies summarized in Table 2 show typical pouch Tmax values in the tens of minutes rather than the very rapid peaks associated with cigarette smoking [37,38,39,40,41].

1. Diffusion through the pouch matrix.
2. Diffusion through saliva.
3. Buccal absorption.

Each of these processes introduces a temporal component that contributes to the observed Tmax. Consequently, products with similar nicotine contents may exhibit dramatically different onset profiles depending on their formulation characteristics.

### 5.1. Area Under the Curve (AUC)

While Cmax and Tmax describe the rate of nicotine delivery, the area under the plasma concentration–time curve (AUC) reflects the total nicotine exposure experienced by the user. AUC is often considered the most comprehensive pharmacokinetic measure because it integrates both the magnitude and duration of nicotine absorption. Two products may produce similar Cmax values while exhibiting very different AUC values if one delivers nicotine rapidly and briefly whereas the other maintains prolonged nicotine release. From a formulation perspective, AUC is influenced by:

- Total nicotine loading.
- Release efficiency.
- Nicotine retention within the pouch.
- Mucosal absorption efficiency.
- Duration of use.

AUC therefore represents the cumulative outcome of all release and transport processes occurring during pouch use.

### 5.2. Elimination Half-Life

Following absorption, nicotine is distributed throughout the body and primarily metabolized in the liver through cytochrome P450-mediated pathways, particularly CYP2A6. The elimination half-life of nicotine in healthy adults is typically approximately two hours, although substantial interindividual variability exists due to genetic, physiological, and behavioral factors. Importantly, elimination half-life is largely independent of pouch formulation. However, the apparent pharmacokinetic profile observed following pouch use reflects the interaction between nicotine elimination and ongoing absorption from the pouch. In slowly releasing formulations, absorption may continue for a substantial portion of the elimination phase, producing complex concentration–time profiles that differ from those observed following rapid delivery systems.

### 5.3. Nicotine Pouches as Dynamic Delivery Systems

A common misconception is that nicotine pharmacokinetics can be predicted directly from nicotine content. In reality, nicotine concentration represents only one variable within a highly dynamic delivery system. The pharmacokinetic profile observed following pouch use emerges from a continuously evolving interplay between hydration, dissolution, pH development, nicotine speciation, diffusion, saliva transport, and mucosal absorption.

Importantly, many of these processes are themselves time-dependent. The local pH may increase gradually as alkaline additives dissolve. The fraction of free-base nicotine may therefore change throughout pouch use. Likewise, nicotine concentrations within the matrix, saliva, and mucosal interface continuously evolve as release and absorption proceed.

The oral cavity should therefore be viewed not as a static equilibrium environment but as a reaction–diffusion system in which the driving forces governing nicotine transport change continuously over time. The growing diversity of nicotine pouch technologies suggests that pharmacokinetic behavior may increasingly be tailored through formulation engineering.

Key design variables include:

- Nicotine source.
- Carrier matrix composition.
- Pore structure.
- Moisture content.
- Water activity.
- pH modifiers.

## 6. Future Directions

Future work should move from descriptive product comparison toward predictive formulation science. The key need is not additional repetition of static nicotine, pH or moisture measurements, but integrated experiments that connect time-resolved hydration, pH, release and permeation with clinical pharmacokinetics. Comparative studies should use matched nicotine dose and, where possible, matched pH, nicotine form, pouch geometry and sensory composition so that the contribution of matrix properties can be isolated.

A second priority is standardization. Pouch-specific methods should be benchmarked against established pharmaceutical tools and should report fluid composition and volume, flow, temperature, mechanical conditions, water activity, matrix density and geometry. Release-model fitting may be useful when the model is validated for the specific pouch system rather than assumed from unrelated dosage forms.

Alternative carrier materials remain scientifically interesting, but the evidence hierarchy must be explicit. Cellulose is established in commercial products; calcium sulfate has direct pouch-specific PK evidence; ion-exchange resins are established in oral nicotine replacement; and mesoporous silicates or zeolites provide mechanistic evidence for nicotine–material interactions. Claims of superior performance or commercial maturity should await independent pouch-specific studies.

Finally, future evaluations should consider tolerability and product-specific risk alongside delivery performance. Relevant endpoints include local irritation, epithelial responses, particle or constituent release into saliva, microbiological stability of high-moisture products, impurity profiles, and the consequences of nicotine stereochemistry and source. These considerations are particularly important when new carrier materials or high-nicotine formulations are introduced.

Another important research area concerns the development of predictive models linking material properties to pharmacokinetic performance. At present, product development often relies heavily on empirical testing and clinical studies. A more rational design approach would establish quantitative relationships between material properties and pharmacokinetic outcomes. Parameters such as water activity, porosity, diffusion coefficients, pH evolution, free-base nicotine fraction, and matrix permeability could potentially be incorporated into predictive models capable of estimating Cmax, Tmax, and AUC prior to clinical evaluation.

Advances in analytical technologies may further accelerate this transition. Real-time imaging, microfluidic oral cavity models, computational fluid dynamics, finite element diffusion simulations, and physiologically based pharmacokinetic (PBPK) modeling may provide increasingly sophisticated tools for understanding and predicting nicotine delivery behavior.

The carrier matrix is an important but still underexplored design variable. Cellulose-based matrices differ substantially in morphology, density and hydration behavior, and calcium sulfate provides a contrasting porous mineral system that has been evaluated. Future comparisons should control confounding formulation variables and include tolerability and safety-related endpoints.

Future progress will depend on standardized dynamic test methods, independent comparative studies and stronger linkage between material properties and clinical pharmacokinetics. Such a framework could improve prediction of onset and duration and support more rigorous comparison of pouch performance.

## 7. Conclusions

Nicotine pouches have evolved beyond simple nicotine-containing consumer products and are more appropriately understood as oral delivery systems. Their performance cannot be inferred from labelled nicotine content alone, because the delivered dose and pharmacokinetic profile emerge from the combined effects of pH, nicotine speciation, hydration, matrix architecture, saliva transport, dissolution, and buccal permeation. Products containing the same nominal nicotine dose may therefore differ substantially in onset, peak exposure, duration of delivery, and the fraction of nicotine extracted during use.

A central implication is that formulation variables should be evaluated dynamically rather than as isolated equilibrium properties. Static measurements of total nicotine, moisture content, or pH remain useful for product description, but they do not fully capture the time-dependent changes that occur after placement in the mouth. Mechanistically informative assessment should combine water activity, hydration kinetics, pH evolution, free-base nicotine fraction, matrix microstructure, nicotine release, and membrane permeation under biorelevant conditions, and should relate these measurements to pharmacokinetic outcomes such as Cmax, Tmax, and AUC.

The carrier matrix is a particularly important and still underexplored design variable. Cellulose is an established commercial pouch carrier, whereas calcium sulfate has been evaluated as a porous mineral alternative in a small clinical study. Other materials, including ion-exchange resins, mesoporous silicates, zeolites, calcium phosphates and polymers, illustrate potential approaches to engineering nicotine–material interactions, but their pouch-specific evidence varies substantially.

Future progress will depend on moving from empirical product comparison toward predictive formulation science. Standardized dynamic test methods, coupled with transport modelling and clinical pharmacokinetics, could establish quantitative relationships between material properties and nicotine exposure. Such a framework would enable rational development of pouches with more predictable onset and duration, improve comparisons between products, and provide a stronger scientific basis for evaluating performance and risk.

## Figures and Tables

**Table 1:** The influence of pH on the amount of free base fraction.

PH	Approximate Free-Base Fraction
6.0	1%
7.0	9%
8.0	50%
8.5	76%
9.0	91%
9.5	97%

**Table 2:** A summary of representative clinical PK studies and illustration of why nominal dose alone is insufficient: exposure varies with product, formulation and use conditions. Values should be compared cautiously because study populations, abstinence periods, use duration and sampling schemes differ.

Study/Product	Dose	Design/Use	Key PK Outcome
McEwan et al. 2022 [37]	6–10 mg	35 adults; randomized crossover	NP Cmax/AUC varied by product; 10 mg Lyft exceeded cigarette exposure in that study.
Azzopardi et al. 2022 [38]	4 mg	34 adults; crossover vs. gum/lozenge	NP Cmax 8.5 ng/mL; AUC0-T 30.6 ng·h/mL; similar to 4 mg lozenge.
Lunell et al. 2020 [36]	3, 6, 8 mg ZYN	Randomized crossover	Dose-dependent extraction and systemic uptake; slower profile than inhaled nicotine.
Chapman et al. 2022 [39]	5.8, 10.1 mg	24 adults; crossover vs. cigarette	Cmax 5.2 and 7.9 ng/mL; Tmax 26 and 22 min.
Franzen et al. 2025 [16]	3, 6 mg calcium sulphate hemihydrate; 6 mg cellulose comparator	12 adults; crossover	Faster early uptake for Ca-sulphate formulations.
Renard et al. 2025 [40]	6, 9, 11.2 mg	30 adults; 30-min use	Cmax 10.92–17.32 ng/mL; Tmax 30–38 min; dose-related exposure.
Azzopardi et al. 2026 [41]	11, 20 mg	Use 10–30 min	Cmax and AUC increased with strength and duration; little nicotine detected in collected saliva.

## Data Availability

No new data were created or analyzed in this study. Data sharing is not applicable to this article.
